# ‘Zero Alcohol Drinks Are Really Complex’: Navigating Substitution and Addition Consumption Issues

**DOI:** 10.1111/dar.70124

**Published:** 2026-03-03

**Authors:** Simone Pettigrew, Bella Sträuli, Asad Yusoff, Paula O'Brien, Michelle Jongenelis, Alexandra Jones, Aimee Brownbill, Fraser Taylor, Jacquie Bowden

**Affiliations:** ^1^ The George Institute for Global Health UNSW Sydney Sydney Australia; ^2^ School of Medicine and Dentistry Griffith University Gold Coast Australia; ^3^ Melbourne Law School The University of Melbourne Melbourne Australia; ^4^ Melbourne Centre for Behaviour Change, Melbourne School of Psychological Sciences The University of Melbourne Melbourne Australia; ^5^ Foundation for Alcohol Research and Education Canberra Australia; ^6^ National Centre for Education and Training on Addiction, Flinders Health and Medical Research Institute Flinders University Adelaide Australia

**Keywords:** advertising, availability, harm reduction, retail, zero alcohol

## Abstract

**Introduction:**

The net benefit of the rapid expansion of the zero alcohol product (ZAP) market has yet to be determined due to the potential for these beverages to produce both positive (substitution) and negative (addition) outcomes. The aims of this study were to: (i) identify factors that can affect substitution consumption; (ii) identify factors that may influence addition consumption; and (iii) explore whether consumers' views on ZAPs are consistent with policy approaches recommended by the World Health Organization.

**Methods:**

Nine online focus groups were conducted with 83 adults residing in three Australian states (51% female). Participants consumed alcohol at least twice monthly.

**Results:**

There was a strong agreement that ZAPs are too expensive but can be useful to facilitate socialising in contexts where alcohol use is expected. Areas of substantial divergence included whether ZAPs taste good and their perceived utility compared to other non‐alcoholic beverages. Concern was expressed for ZAPs constituting a pathway for children to be exposed to products that mimic the taste of alcohol.

**Discussion and Conclusions:**

The ability of the study participants to identify both positive and negative aspects of ZAPs mirrors the tensions between substitution and addition consumption reported in the literature and the resulting policy challenges. While definitive solutions remain elusive in the absence of clear evidence on the impact of ZAPs on alcohol consumption at the population level, the results of the present study suggest there may be community support for policies that are specifically directed at minimising the potential harms of ZAPs for children.

## 1 Introduction

The alcohol market is reaching maturity in many parts of the world, as indicated by the market characteristics of plateauing sales, a proliferation of product categories and brands, intensified advertising and increased price competition [1, 2]. Within this context, alcohol producers are pursuing product diversification and market expansion strategies to sustain growth [3]. A recent manifestation of market maturity has been the rapid evolution of the zero alcohol product (ZAP) category, which is enabling alcohol companies to extend their reach into existing and emerging markets to seek new profit opportunities [4]. ZAPs are defined as products that mimic the taste and appearance of alcoholic beverages, but contain no or very low alcohol content (typically < 0.5% alcohol by volume) [5]. Although strong growth is projected globally over the next few years [6], ZAPs typically represent small proportions of total alcohol sales (e.g., less than 3% in Australia [7] and less than 2% in the United Kingdom [incorporating no and low alcohol products [8]]).

Where ZAPs are used as substitutes for alcoholic beverages, they have the potential to reduce alcohol consumption and associated alcohol‐related harms [9]. Due to the recent emergence of this market, definitive evidence of this substitution effect is lacking. Early research indicates that, in some instances, the use of ZAPs may replace usual alcohol consumption [10, 11], while in other instances, people consume ZAPs in addition to alcohol and thus do not reduce their overall alcohol intake [12]. ‘Addition’ (versus ‘substitution’) use can occur when ZAPs are consumed at times and locations where alcohol would not normally be used (e.g., the evening prior to an early morning commitment or when taking the role of designated driver) and when population sub‐groups that would not normally consume alcohol take the opportunity to drink products that mimic the taste of alcohol (e.g., children and pregnant women).

For ZAPs to provide a net public health benefit, the mechanisms via which they could have adverse consequences need to be addressed [13]. The following possible mechanisms have been identified in the literature: grooming children and adolescents to enjoy the taste of alcohol and develop loyalty to alcohol brands; increased alcohol brand promotion, including in locations where alcohol provision and advertising are currently restricted; causing consumer confusion about the alcohol content of alcohol‐branded products, especially as a result of highly similar packaging design; triggering cravings among those living with alcohol dependence; and blurring the lines for industry participation in the development of public health policies [13, 14, 15, 16, 17, 18, 19]. Strategies recommended by the World Health Organization (WHO) to minimise the potential harms from ZAPs include banning advertising that associates the products with their alcohol brand equivalents, clearly labelling products to avoid consumer confusion, prohibiting the marketing of ZAPs to children and pregnant women, setting guidelines for where ZAPs can be sold, ensuring price differentials based on alcohol content and educating communities to increase awareness of potential risks of ZAPs, especially for vulnerable groups such as children and pregnant women [15]. While the average consumer is unlikely to be aware of or influenced by these policy recommendations, this WHO guidance may influence the normative scope and content of national policy debates over time, ultimately filtering into public opinion [20].

Little is known about whether the concerns expressed in the literature are consistent with consumers' perceptions of the advantages and disadvantages of ZAPs and their likely support for policies designed to minimise potential adverse consequences. The limited research to date suggests ZAPs can be perceived by consumers as healthier alternatives due to the absence of alcohol, but some are concerned they could constitute gateway products for children to become alcohol users [14, 19]. Identified evidence gaps that are stifling the implementation of effective ZAPs harm reduction policies include data on how and why consumers choose to use (or not use) ZAPs, whether ZAPs can normalise alcohol use and how ZAP branding is interpreted by consumers where parent brands have highly similar livery [15].

To contribute to addressing these research gaps, the aims of this exploratory study were to: (i) identify factors that can affect substitution consumption; (ii) identify factors that may influence addition consumption; and (iii) explore whether consumers' views on ZAPs are aligned with the WHO's recommended policy options. The context of the study was Australia, where alcohol products cannot be sold in most supermarkets [21], but ZAPs are able to circumvent this rule, therefore making alcohol branding more visible in locations frequented by children and other vulnerable groups [14]. In addition, there are no formal restrictions on underage purchasing of ZAPs, and ZAP promotion is weakly regulated via a voluntary, industry‐managed advertising code [22].

## 2 Methods

This study was conducted as part of a larger study examining Australians' views on alcohol [23]. Data were collected through nine online focus group discussions involving adult participants who reported consuming alcohol at least twice monthly. Group stratification was applied based on sex, age group, geographic location (metropolitan versus non‐metropolitan) and alcohol intake (within versus exceeding the National Health and Medical Research Council guideline [24]).

The online focus groups were conducted across three Australian states: New South Wales (*n* = 4: 3 metropolitan, 1 non‐metropolitan), Victoria (*n* = 3: 2 metropolitan, 1 non‐metropolitan) and Western Australia (*n* = 2: 1 metropolitan, 1 non‐metropolitan), with participant recruitment facilitated by two ISO‐accredited social research agencies (Chitchat Research and Thinkfield). The sample comprised 83 individuals, with an average of nine participants per group (range: 8–10). There was balanced representation by sex and across three age categories: 18–30 years, 31–50 years and 51+ years (see Table 1). The metropolitan groups were single sex while the non‐metropolitan groups were mixed due to the more challenging recruitment process. Consistent with national population distributions, two‐thirds of participants resided in metropolitan areas [25]. All participants provided written informed consent and received AU$90 remuneration. Ethics approval was granted by a university Human Research Ethics Committee.

**TABLE 1:** Sample profile (*n* = 83).

	*n*	%
Total	83	100
Sex		
Male	41	49
Female	42	51
Age		
18–30 years	28	34
31–50 years	26	31
51+ years	29	35
Location		
Non‐metropolitan area	28	34
Metropolitan area	55	66
Drinking status^a^		
Meets low‐risk guideline	27	33
Exceeds low‐risk guideline	56	68

^a^ National Health and Medical Research Council guideline: no more than 4 drinks on any single occasion and/or no more than 10 drinks per week [24].

Each focus group ran for approximately 90 min. An unstructured interviewing approach was adopted, whereby the topic of ZAPs was discussed when it spontaneously arose during conversations about alcohol. In those instances where ZAPs were not mentioned spontaneously, the topic was raised in very general terms by the moderator towards the end of the session (e.g., ‘So what do we think about zero alcohol products?’). Participants were able to discuss any aspects of ZAPs they considered important or relevant, rather than specific questions being posed. Three of the study authors were present at all the focus groups and shared the moderation role (S.P., B.S. and A.Y.). The sessions were recorded via Microsoft Teams, transcribed verbatim and the transcripts were subsequently imported into NVivo qualitative data management software for coding and analysis.

An inductive analytic strategy was applied that involved iterative development of the coding framework throughout the coding process. The coding was conducted by a single researcher (author S.P.), consistent with best practice in exploratory qualitative research employing emergent coding [26]. Thematic analysis was undertaken using the constant comparative method [27], enabling the identification of patterns and divergences in participants' experiences with and attitudes to ZAPs and their perceptions of appropriate regulatory approaches. Emergent themes were reviewed and refined through collaborative discussion among the research team. Participant quotes are provided below to illustrate major points, with the following key used for the participant descriptors: sex (M, F), age group (18–30, 31–50, 51+ years) and location (metro, regional).

## 3 Results

Only a small minority of the focus group participants reported regular consumption of ZAPs, although many had tried them. Despite low regular usage, the topic of ZAPs was of considerable interest, and there were wide‐ranging discussions about participants' perceptions of the advantages and disadvantages of these products. There were some areas of strong agreement, primarily that ZAPs are too expensive but can be useful to facilitate socialising in contexts where alcohol use is the norm. There were also areas of substantial divergence, such as whether ZAPs taste good and their perceived utility compared to other non‐alcoholic beverages. Ambivalence was common, with many participants expressing both positive and negative views on different aspects of ZAPs. The following quotes provide examples of the ‘double‐edged sword’ nature of ZAPs described by participants and highlight the diverse range of factors influencing use. Males were more likely than females to report using ZAPs and to discuss the trade‐offs between the benefits and disadvantages associated with the evolving ZAPs market.

Obviously it can sort of drive younger kids to drink more alcohol when they actually get to the legal age. But obviously it also works in the opposite direction – if people are trying to cut back on alcohol, it's a bit more convenient being able to get zero alcohol (male, 31‐50 years, regional).

It's kind of a weird balancing act where, on the one hand, if you want a zero alcohol, it has to look somewhat similar, while at the same time it has to be somewhat different, if that makes sense. For example, someone might drink zero alcohol due to some medical condition or something, but they don't want to be left out. So the branding might have to be somewhat similar in that situation. Whereas in others it would be another problem. It's a double‐edged sword is what I'm saying (male, 18‐30 years, metro).

Zero alcohol drinks are really complex. They're probably mostly a social construct … It's an attempt, I think, to try and have the social aspect without the negatives (*of alcohol*) (male, 51+ years, regional).

Addressing Aims 1 and 2, the following sections outline identified issues of relevance to substitution versus addition consumption of ZAPs (summarised in Table 2). The subsequent section addresses Aim 3 by interpreting concerns raised by participants in terms of their alignment with the World Health Organization's policy recommendations for ZAP harm reduction.

**TABLE 2:** Factors influencing substitution and addition use of zero alcohol products.

Perceived barriers to substitution consumption	Perceived facilitators of addition consumption
High cost	Appropriate for more people to use (e.g., drivers, pregnant women)
Poor taste	Can be used in places where alcohol is not permitted (e.g., workplaces)
Lack of intoxication effect	Useful for drinking situations where intoxication is not desired (e.g., weeknights)

*Note:* substitution consumption occurs where alcoholic beverages are replaced with zero alcohol products; addition consumption occurs where zero alcohol products are consumed where alcohol would not otherwise be used [12].

### 3.1 Factors Impeding Use of ZAPs as Substitute Products

Price was by far the most frequently raised perceived disadvantage of ZAPs, followed by unappealing taste. To a lesser extent, the lack of intoxicating effects of ZAPs was also nominated as a barrier to use.

No participants reported considering the cost of ZAPs to be acceptable, and instead current pricing practices were viewed as excessive. Dissatisfaction with ZAPs pricing was typically described in relative terms through comparisons with equivalent alcoholic beverages and/or other non‐alcoholic drinks that are not alcohol flavoured (e.g., soft drinks and juices).

I think they're very overpriced compared to normal alcohol (male, 18‐30 years, metro).

When I go shopping I skip past it, purely because it is more expensive and you could just get soft drink for a cheaper price (female, 18‐30 years, metro).

A main issue appeared to be a lack of understanding of how a product without alcohol could be reasonably expected to cost the same, or even more, compared to one containing alcohol. The following conversation among older women (51+ years) in one of the regional focus groups highlights how price parity with alcohol products was considered inappropriate for ZAPs, especially when lower‐cost products containing alcohol were available in the market.

*Woman 1*: One thing I can't believe is that if you're going to have a beer, say for example, it looks like beer but it's not beer, how come it costs the same price?

*Woman 2*: Now there is half an aisle in Woolworths of zero products and some of the gins and vodkas are $50 and they've got no alcohol in them. I go, “You people are silly because you're just buying flavoured water at $50 a bottle”. Like, crazy.

*Woman 3*: It's not right with the same price. I'm not going to waste my money on that when the wine I drink is quite often cheaper than the no alcohol stuff.

While ZAP price comparisons were made with both alcohol and non‐alcohol alternatives, taste comparisons were almost always only made in relation to equivalent alcohol products. For some, the taste of ZAPs was considered to be close enough to alcohol product counterparts, resulting in a willingness to use them as substitutes on at least some drinking occasions.

I think they're great alternatives because they usually taste the same (male, 18‐30 years, metro).

However, a more common reaction was that expectations for taste parity are not being met. Female participants were usually more emphatic in their accounts of the taste deficiencies of ZAPs.

I tried a non‐alcoholic beer and I didn't like it at all, so my preference would be just to have like a lime and soda or orange juice or something (female, 31‐50 years, metro).

I tasted it – it kind of tasted like wine but it just felt wrong (female 51+ years, regional).

Among those participants prioritising the physiological effects of alcohol, ZAPs were considered futile at best and potentially misleading. A comparison was made with vegan bacon to highlight how distant ZAPs were perceived to be from regular alcohol products.

I don't like it at all, I think it defeats the purpose of having an alcoholic drink. If you want something non‐alcoholic, why not just have water or soft drink? I think myself and also a lot of other people, we like to get that not necessarily drunk feeling, but just a little bit buzzed. That totally defeats the purpose (male, 18‐30 years, metro).

I would not even go near if it says zero alcohol. What's the point? I want alcohol so I wouldn't bother buying it, to be honest. It's just fooling the public (female, 51+ years, metro).

I personally don't see the point in saying that it tastes like alcohol. That is an alcoholic version of vegan bacon. That's not bacon, it just falls into the same category. For me, there's just no point (female, 31‐50 years, regional).

### 3.2 Factors Contributing to the Consumption of ZAPs as Addition Products

Among participants who reported that they or people they knew had used ZAPs, the described nature of the drinking situation often appeared to indicate addition consumption. This addition was due to ZAP use occurring among those who would not normally drink alcohol or in contexts where alcohol use is either not permitted or otherwise not considered appropriate.

In terms of people who would not normally drink alcohol, the two primary groups mentioned were pregnant women and those filling the role of designated drivers. The availability of ZAPs was described as enabling people in these situations to consume alcohol‐flavoured beverages when they would not otherwise have done so.

When I first was pregnant, I really craved beer. It was like a really weird thing for me, and so my husband went out and looked for 0.01 per cent beer, just so that I could have some … obviously I didn't drink any alcohol while I was pregnant (female, 31‐50 years, metro).

When my partner was pregnant, she drank zero alcohol wine through that period of time (male, 31‐50 years, metro).

I have bought them before, like where I've been going to an event and I've been driving so I didn't want to drink, but maybe still wanted the taste of a rum and coke or something (female, 18‐30 years, metro).

I've tried a few zero ones, being a designated driver at times (male, 51+ years, metro).

Specific contexts nominated as being new locations or situations in which ZAPs facilitate the consumption of alcohol‐flavoured beverages were work events and homes. As illustrated in the following quotes, ZAPs can bypass company rules banning the presence of alcohol and they can provide consumption opportunities at other times and locations where the effects of alcohol on cognitive functioning make it unacceptable for use.

I've bought them before from my local (*grocery store*) when I've had a work event where they've asked someone to bring a bottle of non‐alcoholic drink like champagne, because we weren't allowed to drink at the event (female, 18‐30 years, metro).

If it's a school night, I'll often have a non‐alcoholic sparkling instead of an alcoholic drink (female, 31‐50 years, regional).

### 3.3 Alignment Between Consumers' Views of ZAPs and WHO's Proposed Harm Reduction Policies

**FIGURE 1:**
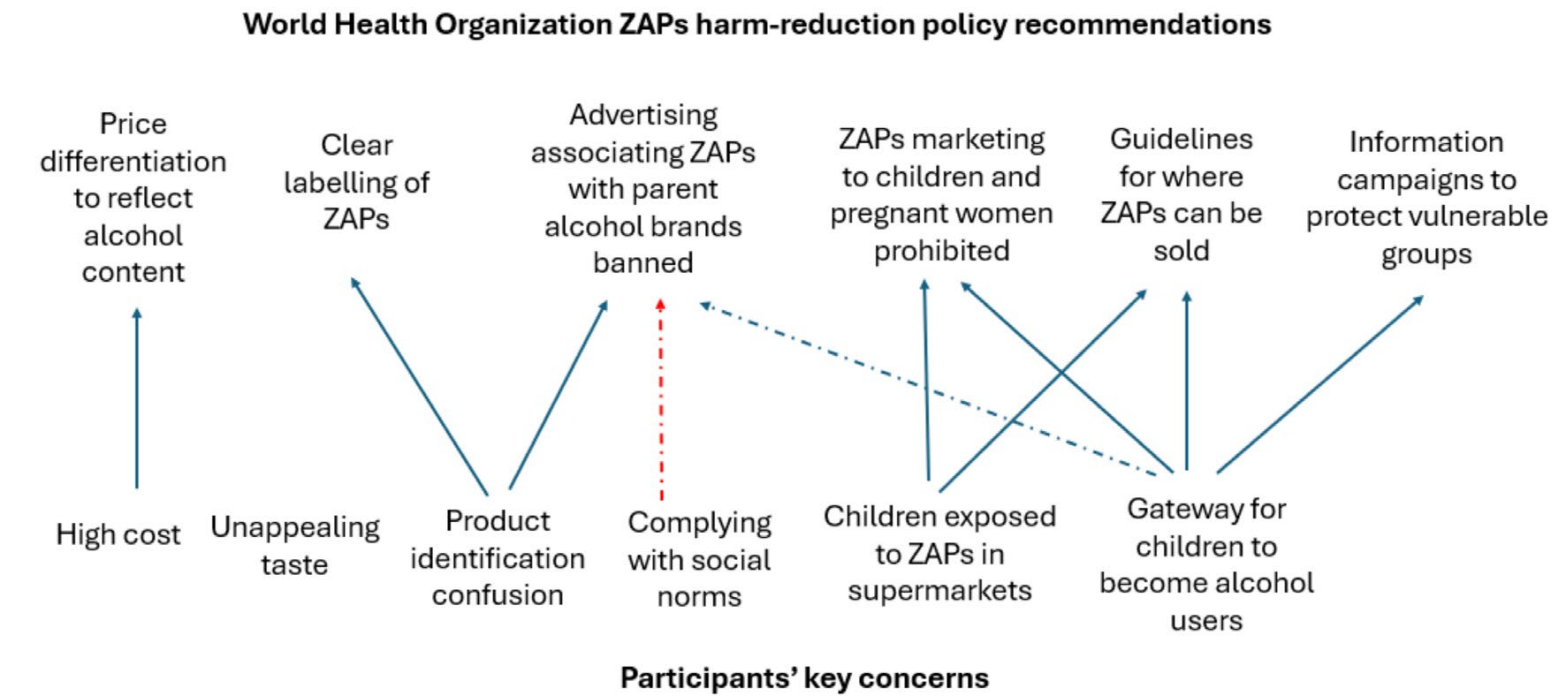
Alignment between identified consumer concerns and harm minimisation recommendations. WHO, World Health Organization; ZAP, zero alcohol product; blue solid lines represent positive relationships, dashed blue line represents a likely relationship and dashed red line represents a negative relationship.

Overall, there was substantial consistency between the concerns expressed by the study participants and recommendations proposed by the WHO to minimise any adverse consequences resulting from the emerging ZAPs market (see Figure 1) [15]. Notably, the strong dissatisfaction with the current price parity between alcohol and zero alcohol products in the Australian market was aligned with the WHO recommendation for alcohol‐flavoured beverages to be priced differentially according to their alcohol content to encourage switching to low and no alcohol products. By comparison, participants' concerns about the taste of ZAPs are not covered by the WHO recommendations, reflecting the focus of the WHO on reducing addiction consumption and its potential consequences for stimulating alcohol use, rather than promoting substitution consumption.

Beyond factors influencing their own consumption, participants discussed a range of other factors they considered to be problematic about ZAPs. In particular, there was concern, especially among older females, about children being newly exposed to ZAPs in supermarkets. This concern was described as being due to increased access to alcohol‐flavoured beverages and branding associated with alcohol products. This exposure to and experience with ZAPs was seen to increase the risk of children's early initiation into alcohol use, which mirrors the rationale underlying the WHO policy recommendations relating to prohibiting ZAPs marketing to children, banning advertising that associates ZAPs with parent alcohol brands, developing guidelines for where ZAPs can be sold and disseminating information campaigns designed to protect vulnerable groups.

I'm concerned because they're available in supermarkets, kids develop a taste for them at a young age. So, they start off with a zero alcohol beer and then they think they might try the alcoholic beer because they've developed a taste for it at a young age, so that's a concern (female, 51+ years, metro).

If we put it on a supermarket shelf, then we are saying that alcohol is fine for kids as well. We're encouraging them to partake rather than discouraging them (female, 51+ years, regional).

I don't really like them when I like see them in the supermarket because they're just on the same shelf as the soft drinks. I feel like that's a bit bad with kids walking along. I get that there's no alcohol in them, but it's still the same brands and everything, and they're made to look like pretty much identical (male, 18‐30 years, metro).

There was some indication that participants believed this was an intentional strategy by the alcohol industry to groom a new generation of alcohol consumers.

I think it's just educating, grooming our children basically to think drinking is not a problem. They start off drinking that and it just leads them into the next step when they turn of age. Well, not even of age, 13, 14. I think it's an issue (female, 51+ years, regional).

Although many participants were concerned about exposure to alcohol branding in supermarkets, there was almost no apparent appreciation of the extent to which children and young people are exposed to ZAPs promotion in other contexts, including mainstream and online advertising and sports sponsorship. This may go some way towards explaining why the advertising restrictions recommended by WHO were not mentioned. A further area of partial misalignment with WHO's recommendations was participants' uniform belief that ZAPs are appropriate for use in pregnancy, which is in contrast to WHO's inclusion of pregnant women as a vulnerable group to be protected from ZAPs marketing due to the products often containing trace amounts of alcohol or alcohol levels higher than stated on the label [28].

Two final topics were raised by participants of relevance to the WHO recommendations. The first was the need for clear labelling of ZAPs to prevent consumer confusion between these products and alcoholic beverages. Due to the highly similar appearance of many ZAPs with their alcohol equivalents, several participants reported finding it difficult to identify ZAPs in alcohol retail stores or their own refrigerators, occasionally leading to incorrect selections.

In a bottle shop they'll be next to each other, and you do have to look at the label to realise that they're actually different (male, 18‐30 years, metro).

It should be clearly labelled because accidentally I bought zero beer once. I've thought, “Well this is just a new kind of beer”. No, it was actually zero alcohol. So I had walked like three blocks, and I promptly turned around and took it back. So I'd like it clearly labelled (female, 51+ years, regional).

Say if I have a bunch of cans in my fridge and I go to hand a can to someone, sometimes my mind won't think which one's the non‐alcoholic one versus the alcoholic one (female, 31‐50 years, metro).

The flip side of this concern, contrary to the WHO recommendation to prevent ZAPs from being promoted in association with their alcohol brand counterparts, was the desire for ZAPs to look like regular alcohol products to facilitate compliance with social norms. Many participants mentioned that the cultural expectation to consume alcohol at social events can make it difficult to abstain without attracting unwanted attention. ZAPs were described as enabling individuals to disguise their non‐drinking status because they look so similar to alcohol products.

The labelling being so similar these days that it's a great alternative – you can seem like you're drinking in a social environment and not get a drop of alcohol, whether you're driving, or you just don't feel like it (male, 18‐30 years, metro).

They're great to have when you don't feel like drinking, but you don't want the comments and questions from people around you (female, 31‐50 years, metro).

I think it takes away a lot of the peer pressure if you're at a social function that people don't tend to notice if you've got an alcohol‐free drink in your hand opposed to a can of soft drink (female, 51+ years, regional).

Some commented that even if others noticed that the product was a ZAP, this was more socially acceptable than consuming other non‐alcoholic beverages such as soft drinks.

I have been at functions where if you suddenly start drinking lemonade, people look at you a little bit funny. Even if it's clearly zero beer, nobody seems to raise an eyebrow and you can get in your car and drive home afterwards (male, 51+ years, regional).

## 4 Discussion

This study identified factors contributing to substitution and addition consumption of ZAPs and explored Australians' views on recommended policies to optimise the outcomes of the emerging ZAPs market. Price and taste were found to be the major determinants of whether the focus group participants reported being willing to select ZAPs as substitutes for alcohol products. This outcome is consistent with the limited prior ZAPs research [29, 30, 31, 32] and the broader food and beverage literature [33, 34], although taste has previously been typically found to dominate price, whereas price issues were found to be paramount here. In the present study, ZAPs were considered to be unsatisfactory on both of these criteria, likely contributing to the low rate of substitution of alcohol products reported within the sample.

Both issues are vexed in terms of the effects of the rapidly growing ZAPs market on public health. Pricing is addressed to some degree in the WHO recommendations through proposed differential taxation based on alcohol content to increase substitution consumption [15]. There is evidence that this strategy can redirect demand towards ZAPs [35]. While this would make ZAPs more affordable compared to full alcohol and low alcohol product alternatives, which is consistent with the expectations of the participants in the present study, a potential downside is that it may also stimulate addition use.

Addressing perceived taste deficiencies presents a different set of challenges. The technical processes for manufacturing ZAPs are evolving, with greater progress made in some alcohol categories (e.g., beer) compared to others (e.g., wine) due to differing organoleptic properties [36]. It is therefore beyond the scope of public health policymakers to intervene in the market to hasten the pace of product development. It seems likely that as greater taste parity with equivalent alcohol products is achieved, both substitution and additional use will increase as consumers find the products more appealing. The increase in addition use could be considerable, given the findings of this and prior research relating to the ability of ZAPs to assist both adults and young people to comply with social norms relating to the consumption of alcohol‐flavoured beverages in social situations [14, 37]. In particular, this finding resonates with the identified practice of attempting to ‘pass’ as a drinker to fit into alcohol‐centred social settings [38]. By mimicking the appearance, packaging and ritual of alcoholic drinks, it appears that ZAPs can lower the interpersonal barriers to abstinence while maintaining the social norms that privilege drinking [32]. The WHO has expressed concern about this addition use due to the resulting reinforcement of an alcohol culture, potentially impeding the success of other initiatives designed to de‐normalise alcohol consumption, especially among young people [15].

An important insight from the present findings is that substitution and addition use of ZAPs appear to be driven by distinct mechanisms. Substitution was most often associated with deliberate harm reduction intent and situational constraints, such as the desire to moderate alcohol intake while maintaining social participation, fulfilling responsibilities (e.g., driving or early commitments), or avoiding intoxication on particular occasions, which is consistent with experimental and field studies demonstrating reduced alcohol consumption when non‐alcoholic alternatives are made available [10, 11]. In contrast, addition use was more closely linked to mechanisms of expanded permissibility and norm compliance, whereby ZAPs enable the consumption of alcohol‐flavoured beverages in new contexts, locations and population groups where alcohol would not ordinarily be consumed [12, 13]. Product characteristics such as alcohol‐like taste, branding and availability are likely to play a central role in both pathways because they support substitution by allowing abstinence without social scrutiny and they facilitate addition by reinforcing alcohol‐related cues, normalising alcohol‐flavoured consumption and lowering barriers for non‐drinkers and younger populations. This highlights the inherent tension in the public health assessment of ZAPs, whereby the same attributes that confer potential benefits for some consumers may also generate population‐level harms if they result in large‐scale addition consumption, a concern that underpins the WHO's precautionary policy recommendations [15].

The participants' concerns relating to children's new exposure to ZAPs in supermarkets were well aligned with the WHO recommendations for restrictions on ZAPs marketing, developing guidelines on where ZAPs can be sold and disseminating information campaigns about potential harms for vulnerable groups. While the participants did not specifically mention the ability of ZAP advertising to increase the reach of alcohol brands to young people, this could be due to prolific alcohol advertising in Australia that sees children already exposed to large amounts of alcohol branding in their day‐to‐day lives [39, 40]. It is possible that, given the stated concerns with the placement of ZAPs in supermarkets, at least some would also support restrictions on the advertising of ZAPs associated with alcohol brands, as per the WHO recommendation. The substantial gap between participants' beliefs about the safety of ZAPs for pregnant women and the WHO's recommendation to prohibit advertising to this group highlights the lack of evidence and consistent guidance on this issue. It also suggests that any policy efforts to reduce the attractiveness of ZAPs to pregnant women would need to include substantial public education to explain the focus on this specific population subgroup.

Finally, the participants' support for clearer ZAPs labelling to avoid confusion is consistent with the WHO recommendation to this effect. This issue is inter‐related with the recommendation relating to banning advertising linking ZAPs with alcohol branding due to the tendency for advertisements to feature product labels. Requiring different label designs would assist in product identification and reduce subconscious associations between alcohol and zero alcohol variants of the same brands. Ensuring ZAPs look substantively different from their alcohol counterparts could thus assist in addressing both issues. This would also serve to reduce the utility of ZAPs as ‘disguise’ products, potentially dampening their reinforcement of pro‐alcohol social norms.

Gender differences emerged in participants' accounts of ZAP use and perceptions. Men were more likely than women to report having used ZAPs and to provide considered evaluations of their relative advantages and disadvantages, often framing these products as tools for moderating alcohol intake while maintaining participation in alcohol‐centred social contexts. In contrast, women more frequently rejected ZAPs on the basis of taste and perceived pointlessness, instead preferring non‐alcohol‐flavoured alternatives such as soft drinks or juice when abstaining. These differences may reflect broader gendered norms around alcohol consumption, whereby men experience stronger expectations to appear as drinkers in social settings while women may face fewer social sanctions for visibly abstaining [41]. This may make ZAPs more appealing for men as a means of preserving social conformity without intoxication, while ZAPs may have lower functionality for women with less need for ‘disguise’ products (an exception being during pregnancy [42]). Gendered social roles were also evident in the heightened concern expressed by older women regarding children's exposure to ZAPs in supermarkets, suggesting that caregiving and protective norms may shape how potential harms of ZAPs are perceived and prioritised. These differences suggest that WHO‐recommended policy approaches to minimise addition use and protect vulnerable groups may have differential impacts across men and women, underscoring the importance of considering gendered drinking norms and social roles when designing and implementing ZAP‐related regulation and public education initiatives.

The primary limitation of this study was the modest sample resulting from the exploratory qualitative approach. While this enabled issues of most relevance to the participants to emerge during discussions, the findings cannot be considered generalisable to the broader population of alcohol users in Australia, nor to individuals residing in other countries. Further research with representative samples is required to assess the extent to which the present results are indicative of broader trends. The unstructured nature of the conversations relating to ZAPs could also have resulted in other issues of importance failing to arise. In particular, participants were not specifically asked their views on the WHO's policy recommendations, and it is possible that a more direct approach could have yielded a somewhat different interpretation.

In conclusion, the study findings highlight areas that may need to be addressed to enhance the substitution benefits of ZAPs while minimising any adverse consequences. Although definitive solutions remain elusive in the absence of clear evidence on the impact of ZAPs on alcohol consumption at the population level, it appears there may be community support for policies that are specifically directed at addressing the potential harms of ZAPs for children.

## Author Contributions

Conceptualisation: S.P. Methodology: S.P. Formal analysis: S.P. Investigation: S.P., B.S., A.Y. Writing – original draft: S.P. Writing – review and editing: B.S., A.Y., P.O'B., M.J., A.J., A.B., F.T., J.B. Funding acquisition: S.P., P.O'B., M.J., A.J., A.B., F.T., J.B.

## Funding

This study was funded by a National Health and Medical Research Council grant #2021186.

## Conflicts of Interest

The authors declare no conflicts of interest.

## Data Availability

The authors have nothing to report.
